# First Clinical Experience with a Carbon Fibre Reinforced PEEK Composite Plating System for Anterior Cervical Discectomy and Fusion

**DOI:** 10.3390/jfb10030029

**Published:** 2019-07-02

**Authors:** Helena Milavec, Christoph Kellner, Nivetha Ravikumar, Christoph E. Albers, Till Lerch, Sven Hoppe, Moritz C. Deml, Sebastian F. Bigdon, Naresh Kumar, Lorin M. Benneker

**Affiliations:** 1 Department of Orthopaedic Surgery, Spine Unit, Inselspital, Bern University Hospital, University of Bern, 3010 Bern, Switzerland; 2 Department of Orthopaedic Surgery, National University Health System (NUHS)–Tower Block, Level 11, 1E Kent Ridge Road, Singapore 119228, Singapore

**Keywords:** carbon, PEEK, ACDF, CFR-PEEK, cervical spine, trauma, degenerative, tumour

## Abstract

Carbon fibre reinforced polyether ether ketone (CFR-PEEK) is a suitable material to replace metal implants in orthopaedic surgery. The radiolucency of CFR-PEEK allows an optimal visualisation of the bone and soft tissue structures. We aimed to assess the performance and radiological and clinical outcomes of anterior cervical discectomy and fusion (ACDF) with CFR-PEEK anterior cervical plating (ACP) under first use clinical conditions. We retrospectively studied the prospectively-collected data of 42 patients who underwent ACDF with CFR-PEEK ACP between 2011 and 2016. We assessed clinical outcome (Odom’s criteria, complications) and radiological parameters (global and segmental cervical lordosis, Bridwell score for fusion, adjacent segment degeneration) preoperatively, immediately post-operatively, and after a 12-month follow-up period. Patients’ satisfaction was excellent, good, fair, and poor in 12, 19, 3, and 1 patients, respectively. Two patients developed dysphagia. No hardware failure occurred. Compared with preoperative radiographs, we observed a gain of global cervical lordosis and segmental lordosis (7.4 ± 10.1 and 5.6 ± 7.1 degrees, respectively) at the 12-month follow-up. Bridwell IF grades I, II, and III were observed in 22, 6, and 7 patients, respectively. The 12-month adjacent segment degeneration-free and adjacent segment disease-free survival rates were 93.1% and 96.3%, respectively. We observed a dysphagia rate of 5.7% and a reoperation rate of 4.8%. In conclusion, CFR-PEEK ACP shows positive outcomes in terms of implant safety, restoration of cervical lordosis, and functional recovery, and is suitable for ACDF.

## 1. Introduction

Anterior cervical discectomy and fusion (ACDF) constitutes a well-established treatment modality in cervical spine surgery, with good clinical outcomes on long-term follow-up for mono-segmental and multi-segmental cervical fusion [1]. The importance of having the right sagittal balance of the cervical spine and its impact on quality of life [2], as well as the development of adjacent segment degeneration (ASDeg) and disease (ASDis) [3] following ACDF, has been stressed upon in literature. ACDF with or without anterior plate fixation are both accepted surgical techniques. The addition of the anterior plate, however, may favour the preservation of segmental lordosis [4]. Currently, the most popular orthopaedic materials for anterior cervical plating (ACP) systems are metals such as titanium and its alloys [5].

In the late 1990s, polyether ether ketone (PEEK) emerged as the leading high-performance thermoplastic material for replacing metal implants, especially in orthopaedics and trauma. PEEK is a semi-crystalline polymer with a good combination of strength, stiffness, toughness, and environmental resistance. It is exceedingly unreactive and inherently resistant to chemical, thermal, and post-irradiation degradation [5,6]. Compared with stainless steel and titanium, an implant made of PEEK has clear benefits in terms of thermo-resistance, lightweight, favourable radiological features, and the fact that it allows for good biocompatibility [6]. Titanium coating or carbon fibre reinforcement (CFR) have further improved the properties of PEEK [7]. Owing to the chemical stability and thermal resistance of CFR-PEEK, it sustains common sterilisation methods for medical devices such as steam sterilisation and gamma radiation. CFR-PEEK has gained increased acceptance as an alternative to metallic biomaterials owing to its high-performance and radiolucency, and a recent systematic review strongly supports its use [8].

One of the advantages of CFR-PEEK over metal implants is the elimination of imaging artefacts. The radiolucency of CFR-PEEK allows optimal visualisation of bone fusion, and it is compatible with both computed tomography and magnetic resonance imaging technologies. The radiolucency of implants made of CFR-PEEK facilitates the management of spinal tumours in terms of post-operative imaging, radiation therapy planning, and follow-up for tumour recurrence [9,10]. Tedesco et al. have shown that the absence of image artefacts together with significantly lower dose perturbations may improve the treatment accuracy in radiotherapy [10]. Compared with metallic implants, CFR-PEEK allows an optimal visualisation of the bone and soft tissue structures, and may thus aid in identifying early tumour recurrence [10].

A novel and unique CFR-PEEK-based ACP system has recently been developed (icotec ag, Altstaetten, Switzerland). According to the anterior cervical plate nomenclature described by ‘The Cervical Spine Study Group’, this ACP system has a restricted backout plate (unicortical locked screws) [11].

This study with a level of evidence of IV, aimed at investigating (1) the efficacy and safety assessed by complications and patients self-reported outcomes, and (2) the clinical assessment and (3) radiographic outcome as assessed by segmental and global cervical lordosis, Bridwell score for fusion, and ASDeg in patients undergoing ACDF procedures using the CFR-PEEK ACP system. 

## 2. Materials and Methods

A retrospective analysis of prospectively-collected data was carried out at a single tertiary care institution. Ethical approval was granted by the Ethics Commission of the Canton of Bern, before the commencement of the study. We enrolled 42 consecutive patients aged between 18 and 90 years who underwent ACDF with icotec CFR-PEEK ACP between 2011 and 2016. The indication for surgery was trauma (fracture and/or discoligamentous injury), degenerative disease (disc herniation and/or spinal stenosis), and primary tumour or metastatic disease. We collected patient demographics and surgical details. We assessed clinical and radiological parameters preoperatively, in the immediate post-operative period (median (IQR): 2 (1–3) days), and at 6 and 12 months after surgery. We compared the radiological parameters between the trauma and degeneration subgroups, as well as between the mono-segmental and multi-segmental(bi-/tri-segmental) ACDF groups. The senior author performed all surgeries and used the standard anterior approach. After segmental decompression, the intervertebral disc was replaced. We used allograft spacer cages in 36 patients, PEEK cages (1× Medtronic Cornerstone, 1× Icotec cervical cage) in 2 patients, an expandable corpectomy device (ECD) in 2 patients, and a structural iliac crest bone autograft in 1 patient. In one patient suffering from a secondary, we did not use any cage to achieve cervical fusion. We never used bone morphogenetic proteins, demineralized bone matrix, or allogeneic bone grafts.

### 2.1. Clinical and Radiological Outcome Measures

**Figure 1:**
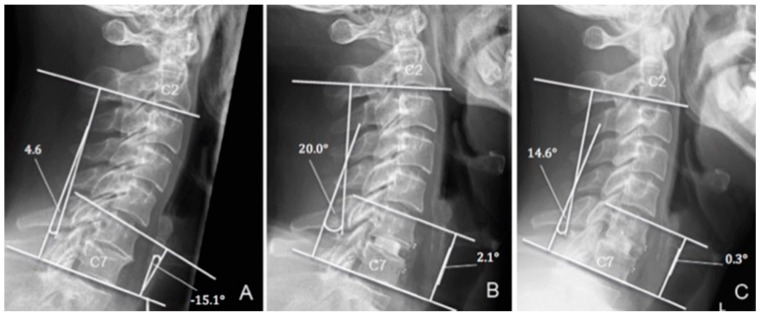
Sagittal segmental alignment (SSA) and sagittal alignment of the cervical spine (SACS) in a 45-year-old patient suffering from trauma. (**A**) preoperative (−15.1° and 4.6°, respectively), (**B**) early postoperative (2.1° and 20.0°, respectively), and (**C**) at 12-month follow up (0.3° and 14.6°, respectively).

The clinical outcome was measured according to Odom’s criteria [12]. Erect cervical spine radiographs were obtained preoperatively and at each follow-up. Radiologically, the segmental alignment of the operated segment(s) and the global sagittal profile of the sub-axial cervical spine were measured pre-operatively and at each follow-up, using the ‘sagittal segmental alignment’ (SSA) and ’sagittal alignment of the cervical spine’ (SACS), respectively (Figure 1). Faldini et al. defined the SSA as the angle between the line parallel to the upper vertebral endplate of the proximal vertebra to the involved disc space and the line parallel to the lower vertebral endplate of the underlying vertebra [13]. SACS was defined as the angle between a line parallel to the inferior endplate of C2 and C7 [14]. Lordotic and kyphotic angles were considered to be positive (+) and negative (−), respectively. Potential screw loosening was detected using the ε_2_-angle according to Aghayev et al. [15]. An ε_2_-angle change of 2° between the immediate and the 12 month-post-operative time-points was set as the cut-off value for screw loosening [15]. We investigated the occurrence of ASDeg in one segment above and below the operated level and applied the ‘composite radiographic score’ (CRS), previously described by Benneker et al. [16]. To account for pre-existing degenerations, we assessed delta (∆) CRS by comparing the CRS at 12-month follow-up with the score calculated in the immediately postoperative period. We used the early immediately postoperative data instead of preoperative data, as part of the preoperative imaging was performed by referring physicians. Hence, these time points may vary more than the postoperatively taken images during hospitalisation, and thus may hinder a precise timing of the 12-month follow-up period. Interbody fusion (IF) was evaluated at the 12-month follow up utilizing the Bridwell criteria [17].

### 2.2. Statistical Analysis

Continuous variables among the study groups ‘trauma’ and ‘degeneration’ were compared with the unpaired Student’s t-test. For SACS and SSA, comparison was made among three time-points—the preoperative values to early postoperative values, preoperative values to the 12-month follow up, and early postoperative values to the 12-month follow up—using the paired Student’s t-test. The proportion of patients who did not develop ASDeg and ASDis were analysed using the Kaplan–Meier product limit method, and 95% confidence intervals were obtained per the Greenwood method. Median follow-up duration was calculated using the reverse Kaplan–Meier method [18]. The association between cervical pain and age ≥55 or sex was tested using $\chi^{2}$ tests. Statistical analyses were performed using Winstat software (R. Fitch Software, Bad Krozingen, Germany, Version 2012.1) and Stata (StataCorp, Version 13.0).

## 3. Results

A total of 42 patients were enrolled, including 6 (14.3%) females and 36 (85.7%) males. The average age at surgery was 49.7 ± 15 years (range, 18–80). The overall median follow up for the cohort was 16.8 months (IQR: 16.8–35.5). Table 1 summarises baseline characteristics and indications for surgery. The majority of patients had mono-segmental ACDF (n = 25, 59.5%), followed by bi-segmental ACDF (n = 16, 38.1%) and tri-segmental ACDF (n = 1, 2.4%). One (2.4%) patient had two mono-segmental ACDFs at non-contiguous levels. The most affected levels for mono-segmental and bi-segmental ACDF were C6–C7 (n = 12, 28.6%) and C5–C7 (n = 5, 11.9%), respectively. Thirty-five (83.3%) patients, 5 (14.3%) females and 30 (85.7%) males, were available for assessment of SACS and SSA. Of seven patients who were unable to achieve the long-term follow up, two had early revision surgeries requiring plate removal or posterior stabilisation, four were tourists, and one suffered from psychiatric disease.

### 3.1. Clinical Outcomes

Of 35 available patients, 12 (34.3%), 19 (54.3%), 3 (8.6%), and 1 (2.9%) reported excellent, good, fair, and poor satisfaction, respectively, according to Odom’s criteria [12]. Ten (28.6%) patients suffered from occasional cervical pain. Two (5.7%) patients experienced post-operative dysphagia, while two (5.7%) patients had implant-related complications (Table 2). Neither gender (*p* = 0.777) nor age (*p* = 0.735) were found to be risk factors for post-operative cervical pain.

### 3.2. Radiological Outcomes

**Figure 2:**
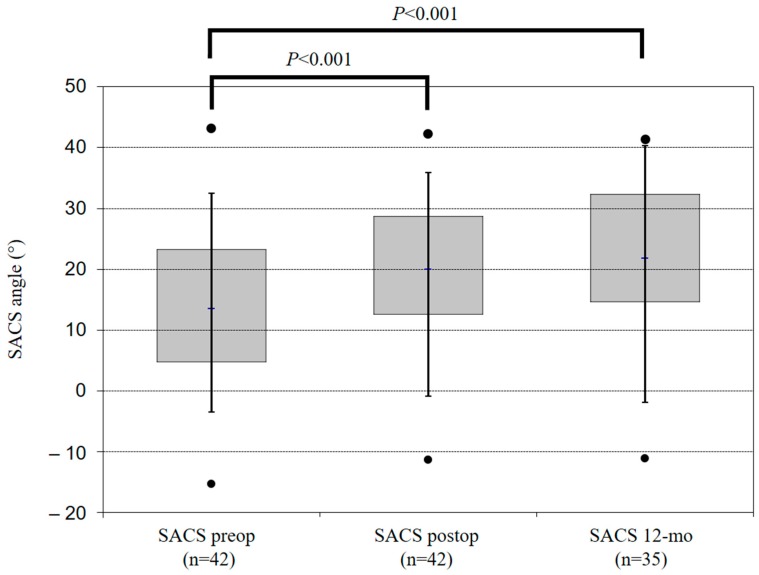
These boxplots show the SACS angle in different points in time (n = 42). Positive values of the SACS angle represent lordosis, negative values represent kyphosis.

**Figure 3:**
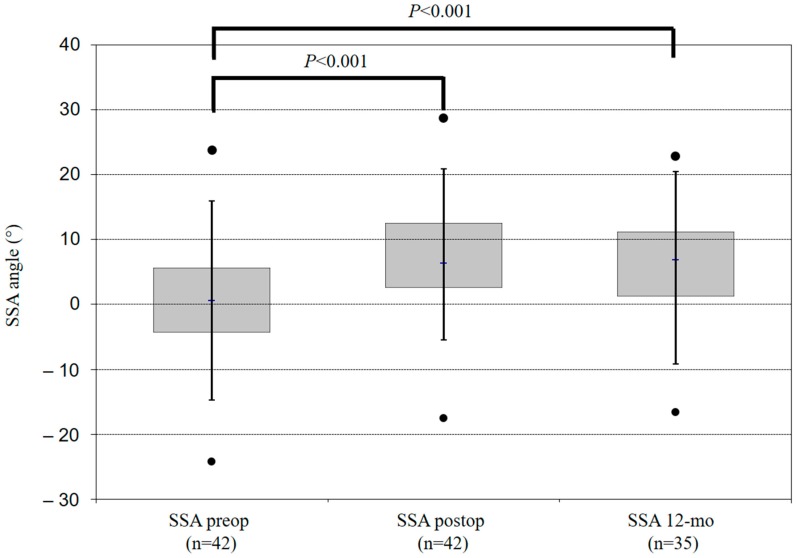
These boxplots show the mean SSA angle in different points in time for all (n = 42). Positive values of the SSA angle represent lordosis, negative values represent kyphosis.

Optimal implant placement was visualised on the immediate postoperative film in all patients except one. At the 12-month assessment, no implant failure occurred due to breakage of either the plate or the screws. Overall, ACDF led to an improvement of both SACS and SSA at the 12-month follow up (Table 3). Compared with preoperative radiographs, early postoperative radiographs showed a mean increase in cervical and segmental lordosis of 6.1 ± 9.1 degrees (*p* < 0.05) and 6.6 ± 6.1 degrees (*p* < 0.005) for SACS and SSA, respectively (Figure 2 and Figure 3). At the 12-month follow up, compared with early postoperative values, overall SACS displayed a statistically non-significant increase of 0.7 ± 8.4 degrees (*p* = 0.643) and SSA showed a non-significant decrease of previously-gained lordosis of 1.1 (±3.9, *p* = 0.094) degrees. Compared with preoperative radiographs, we observed a gain of cervical and segmental lordosis (SACS: 7.4 ± 10.1 and SSA: 5.6 ± 7.1 degrees) at the 12-month follow up (Table 3).

We compared the change in the radiological parameters at the three time-points of interest between the trauma and the degeneration groups. This similar analysis was also carried out comparing mono-segmental and multi-segmental ACDF groups. No statistically significant differences were delineated when comparing the change in radiological parameters between these groups (Table 4).

Radiographic ASDeg occurred in 5 of 35 patients over the course of the follow up. CRS changed from 0 to 1, 0 to 3, 2 to 3, 3 to 4, and 4 to 5, respectively. In one case, ASDeg occurred in the segment below (C3/4) and in four cases in the segment above (2× C4/5, 2× C5/6) the level of ACDF.

**Figure 4:**
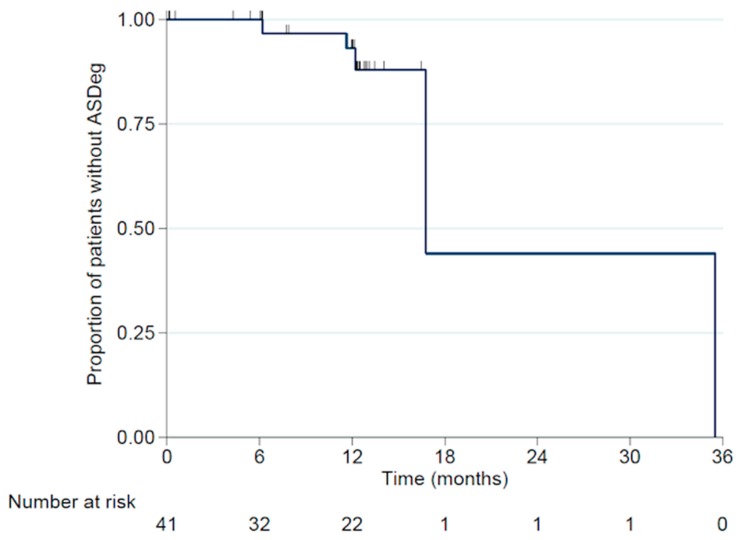
Adjacent segment degeneration (ASDeg)-free survival rate.

**Figure 5:**
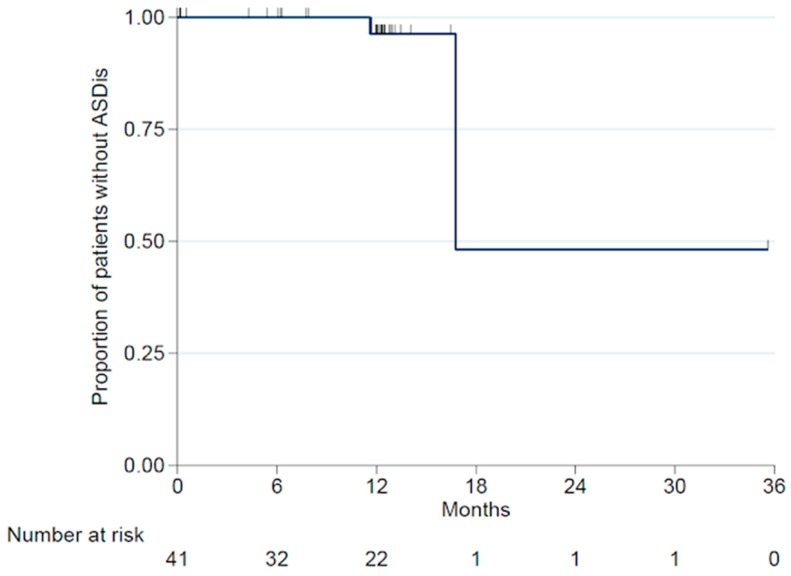
Adjacent segment disease (ASDis)-free survival rate.

The indications for primary surgery in these five patients were trauma (n = 3) and degeneration (n = 2). The 6-month and 12-month ASDeg-free survival rates were 100% and 93.1% (95% CI:75.1-98.2%), respectively (Figure 4). Of these five patients with ASDeg, two became symptomatic (i.e., ASDis) at 11.6 months and 16.8 months post-surgery, resulting in a 6-month and 12-month ASDis-free survival rate of 100% and 96.3% (95% CI: 76.5–99.5%), respectively (Figure 5). Both patients who developed ASDis were from the degenerative disease subgroup.

Of 35 patients available, complete IF (grade I) was observed in 22 (62.9%) patients; grade II in 6 (17.1%), and grade III in 7 (20.0%). Grade IV was not observed. We have depicted the fusion rates based on indication and number of levels in Supplementary Table S1.

### 3.3. Complications

Two of 42 (4.8%) patients developed severe complications requiring revision surgery. One suffered from severe dysphagia two weeks after index surgery, presumably as a result of poor plate positioning, and underwent revision with plate removal. The second patient had a cage subsidence in the tumour-involved bone with a screw pullout, and subsequently underwent revision with posterior stabilisation. Another patient encountered screw loosening with an ε_2_-angle change of 6.2 degrees at 6 months post-operatively. However, owing to a good clinical outcome and radiologically-proven IF, revision surgery was not required. No cases of recurrent nerve palsy, infection, or death occurred in relation to the procedure, and there were no instances of hardware failures (Table 2).

## 4. Discussion

**Figure 6:**
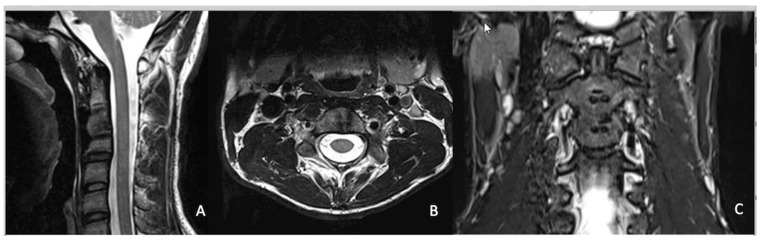
Cervical MRI of a twenty-year-old patient after trauma and consecutive ACDF with CFR-PEEK plate level C2/3. No artefacts can be seen. (**A**) Sagittal plane, (**B**) axial plane, and (**C**) frontal plane.

In the present study, we found that a CFR-PEEK ACP is associated with positive clinical and radiological outcomes and can be used in the vast majority of cervical spine pathologies. To the best of our knowledge, this is the first study to report outcomes of a CFR-PEEK ACP system for ACDF. Presently, many implants for anterior cervical plating are available on the market [19]. The most popular materials for ACP systems are titanium and its alloys [5]. Nevertheless, their relatively high elastic moduli cause stress shielding, thereby leading to adjacent bone resorption [5]. In addition, they are radio opaque [5] and thus may hinder radiological interpretation. New biomaterials have been developed to improve the biocompatibility and offer superior features including reduction of postoperative radiological artefacts [5,6]. CFR-PEEK ACP produces minimal artefacts in post-operative MRI (Figure 6). The radiolucency of the CFR-PEEK ACP system is of great value to tumour patients [9,10]. In a recent study of 34 tumour patients, Boriani et al. found that the clinical use of CFR-PEEK composite implants seemed to be at least comparable with the commonly used titanium implants regarding intraoperative complications, stability at weight bearing, and at functional recovery [20].

Follow-up studies assessing the advantages of CFR-PEEK ACP system in terms of post-operative imaging and radiotherapy are subjects of ongoing research.

In our cohort, complete or good fusion (grades I and II) was achieved in 28 of 35 patients (80.0%), while the remainder (n = 7; 20%) had incomplete fusion (grade III). Six patients with grade III fusion maintained their postoperative implant position with no evidence of implant failure and exhibited good clinical outcomes. One patient had a follow-up duration of four months only. We surmise that given time, this patient would achieve good fusion. Nevertheless, the fusion rate in our study seems rather high as compared with the existing literature [21,22]. A systematic review by Miller et al. [21] reported radiographic fusion rates to be 91% with allograft and autograft and 97.1% with cage replacement. Lately, Oshina et al. reported in their review a mean one-year fusion rate of 90.2% [22]. However, Oshina et al. found that criteria are subjectively determined because of the lack of an objective scale to quantify the findings on plain radiographs. One (2.9%) of the patients with grade III fusion developed screw loosening, and is likely to develop non-union. A recent meta-analysis by Shriver et al. including eleven studies with a follow-up of 12 to 24 months reported a pooled pseudoarthrosis rate of 3.1% [23]. This compares well with our pseudoarthrosis rate of 2.9%. It is observed that the definition of pseudoarthrosis varied significantly within the literature [22,23,24]. We stratified our patients with IF grade III for possible risk factors and could not identify any predisposing risk factors in our study (Table S2) [25,26,27].

Compared with preoperative data, overall segmental lordosis and overall global cervical lordosis (C2–C7) significantly increased postoperatively by 6.1 degrees and 7.4 degrees, respectively. These results are consistent with those reported in the current literature [1,2,3]. The concern of gradual loss of restored sagittal alignment over the course of time has been well discussed. In our results, SSA decreased by 1.1 degree (±3.9 degrees), which was not statistically significant (*p* = 0.094). The global cervical lordosis increased from early to late postoperative follow up by 0.7 ± 8.4 degrees (*p* = 0.643), which was not statistically significant.

ASDeg/Dis remains a common issue after spinal fusion. In our study, the 12-month adjacent segment degeneration (ASDeg)-free and adjacent segment disease (ASDis)-free survival rates were 93.1% and 96.3%, respectively. No trauma patient developed ASDis and no revisions were required for ASDis. Hilibrand et al. reported the annual incidence of symptomatic adjacent-level disc degeneration after fusion to be 2.9% [28], and a recent review by Shriver et al. found that the incidence of ASDeg and ASDis was 8.3% and 0.9%, respectively [29].

We found no correlation between age or sex and post-operative cervical pain, corroborating a recent study that reported that age, gender, and the number of levels treated are unrelated to long-term outcomes [30]. In our study, the overall complication rate including dysphagia was 11.4% (n = 4/35). Our study showed lower rates (5.7%) of postoperative dysphagia as compared with the wide range of values reported in the existing literature [31,32,33]. In a meta-analysis by Shriver et al., an overall dysphagia rate of 8.5% was reported [32]. Given that the thickness of the plate is comparable to that of commercial titanium plates, the low rate of dysphagia may imply either good biocompatibility of the CFR-PEEK ACP system or expertise of the surgeon.

We observed a reoperation rate of 4.8% (2/42). The first patient suffered from severe post-operative dysphagia and required early plate removal. This complication occurred in the first of our patients treated with the CFR-PEEK ACP system, which may reflect the learning curve of the surgeon, given that the plate was placed at a 3 mm distance from the vertebrae. The second case was a cage subsidence observed after corpectomy in a cancer patient owing to poor bone quality secondary to tumour involvement, thereby necessitating additional posterior stabilization. There were no procedure-related infections, death, recurrent laryngeal nerve palsy, Horner syndrome, or cerebrospinal fluid leakage. In our cohort, no instrument failure occurred, consistent with existing literature on ACDF [31,33].

### Limitations

Interpretation of this study should be placed in the context of the inherent weaknesses and limitations of a retrospective analysis; however, data for this study were prospectively-collected according to pre-specified standardised forms. Moreover, this study lacks a control group with ‘conventional’ implants. A thorough literature review was performed for comparison with published data. Unfortunately, we did not have flexion-extension views or CT scan at the 12-month follow up to better quantify our fusion rates. The small number of patients, particularly tumour patients, limits the statistical power of our study. Furthermore, our relatively short follow-up duration because of the novelty of this ACP system hinders arriving at a definite conclusion about ASDeg/ASDis.

## 5. Conclusions

To the best of our knowledge, this is the first study dealing with the use of CFR-PEEK ACP system for ACDF. The CFR-PEEK ACP system exhibits positive outcomes in terms of implant/construct acceptance, safety and stability, as well as functional recovery. We also demonstrated a persistent improvement of global and segmental cervical lordosis. Furthermore, we observed a lower rate of dysphagia as compared with the existing literature. We suggest that the CFR-PEEK ACP system is suitable for ACDF in trauma and degenerative disease. Our study paves the path for future comparative studies with a larger sample size and longer follow-up duration, outlining the suggested benefits of radiolucency and absence of imaging artefacts, ultimately allowing for more precise radiological follow up. In particular, larger series of tumour patients are required to confirm the data on tumours.

## Figures and Tables

**Table 1:** Baseline characteristics and demographics. M—male; F—female; ACDF—anterior cervical discectomy and fusion.

Age (Median (IQR))	Gender (M:F)	Indication	Number of Cases Undergoing ACDF (%)
51.9 (44.5 to 58.6)	36:6	Trauma	23 (54.8%)
		Degeneration	16 (38.1%)
		Tumour	3 (7.1%)
		Total	42 (100%)

**Table 2:** Complications.

Complications	Number of Cases (%)
Dysphagia	2 (5.7%)
Screw loosening	1 (2.9%)
Cage subsidence	1 (2.9%)
Infections	0 (0.0%)
Recurrent nerve palsy	0 (0.0%)
Hardware failure	0 (0.0%)
Death related to procedure	0 (0.0%)

**Table 3:** Mean sagittal alignment of the cervical spine (SACS) and sagittal segmental alignment (SSA) at three different time-points and mean angle-changes from preoperative to postoperative and from postoperative to 12-month follow up.

Group	Angle	Time Point of Measurement					Mean Change of SSA and SACS Between Two Measurements					
		Preop (n = 42) *(±SD)*	Preop (n = 35) *(±SD)*	Postop (n = 42) *(±SD)*	Postop (n = 35) *(±D)*	12-Mo *(±SD)*	Preop to Post Op (Degrees) *(±SD)*	*p*-Value	Preop to 12-Mo (Degrees) *(±SD)*	*p*-Value	Postop to 12-Mo (Degrees) *(±SD)*	*p*-Value
Overall	SACS	13.5 *(±11.7)*	14.1 *(±12.3)*	19.6 *(±11.7)*	20.8 *(±11.3)*	21.5 *(±12.3)*	+6.1 *(±9.1)*	<0.001	+7.4 *(±10.1)*	<0.001	+0.7 *(±8.4)*	0.643
	SSA	0.5 *(±8.9)*	1.1 *(±9.4)*	7.1 (±7.7)	7.8 (±7.9)	6.7 (±7.8)	+6.6 *(±6.1)*	<0.001	+5.6 *(±7.1)*	<0.001	−1.1 *(±3.9)*	0.094
Trauma	SACS	12.7 *(±11.5)*	12.9 *(±12.2)*	20.1 *(±10.1)*	20.5 *(±10.9)*	20.6 *(±11.9)*	+7.4 *(±8.5)*	<0.001	+7.7 *(±12.5)*	<0.05	+0.1 *(±7.9)*	0.945
	SSA	−2.6 *(±9.5)*	−2.5 *(±10.3)*	5.3 *(±6.8)*	5.2 *(±7.3)*	4.0 *(±7.2)*	+7.9 *(±6.8)*	<0.001	+6.5 *(±8.6)*	<0.05	−1.2 *(±4.3)*	0.249
Degeneration	SACS	14.3 *(±13.4)*	15.4 *(±13.1)*	18.6 *(±14.3)*	20.6 *(±12.2)*	21.4 *(±12.7)*	+4.3 *(±10.0)*	0.111	+6.0 *(±5.6)*	<0.05	+0.8 *(±9.3)*	0.728
	SSA	5.4 *(±6.0)*	5.8 *(±6.1)*	10.6 *(±7.9)*	11.3 *(±7.7)*	10.1 *(±7.5)*	+5.2 *(±5.3)*	<0.05	+4.3 *(±4.8)*	<0.05	−1.2 *(±3.5)*	0.202
Monosegmental	SACS	11.8 *(±12.9)*	12.5 *(±13.4)*	18.1 *(±13.1)*	19.7 *(±12.6)*	20.7 *(±13.4)*	+6.3 *(±9.7)*	<0.05	+8.2 *(±10.4)*	<0.05	+1.0 *(±7.6)*	0.553
	SSA	−0.9 *(±9.4)*	−0.6 *(±10.1)*	5.2 *(±7.0)*	5.4 *(±7.6)*	4.3 *(±7.4)*	+6.1 *(±6.9)*	<0.05	+4.9 *(±8.3)*	<0.05	−1.1 *(±4.2)*	0.247
Bi-/Trisegmental	SACS	15.8 *(±10.1)*	16.3 *(±10.7)*	21.7 *(±9.5)*	22.4 *(±9.55)*	22.6 *(±11.1)*	+5.9 *(±8.5)*	<0.05	+6.3 *(±10.1)*	<0.05	+0.2 *(±9.6)*	0.943
	SSA	2.5 *(±8.3)*	3.4 *(±8.2)*	9.7 *(±8.1)*	11.2 *(±7.1)*	10.1 *(±7.3)*	+7.2 *(±4.9)*	<0.001	+6.7 *(±5.1)*	<0.001	−1.1 *(±3.5)*	0.234

SD = standard deviation. All angle- and mean change-values in degrees. Angle-values are considered positive in lordosis and negative in kyphosis. Significant *p*-values are in bold.

**Table 4:** Comparison of SACS and SSA between the indication groups at different points of time.

Groups	Angle	Preoperative to Postoperative	Postoperative to 12-Month Follow Up
		*p*-Values	*p*-Values
Trauma vs. Degeneration	SACS	0.302	0.808
	SSA	0.185	0.991
Monosegmental vs. Bi-/trisegmental	SACS	0.857	0.773
	SSA	0.612	0.990

There are no significant differences in the mean gain of lordosis between the indication groups.

## Supplementary Materials

The following are available online at https://www.mdpi.com/2079-4983/10/3/29/s1, Table S1: Fusion rates based on indication and number of levels; Table S2: Possible predisposing factors for nonunion.
