# Supplementary material for: First Clinical Experience with a Carbon Fibre Reinforced PEEK Composite Plating System for Anterior Cervical Discectomy and Fusion
Source: J Funct Biomater. 2019 Jul 2;10(3):29. doi: 10.3390/jfb10030029 (PMC6787668; doi:10.3390/jfb10030029)
Supplement: Supplementary file 1 [file jfb-10-00029-s001.pdf]

First Clinical Experience with a Carbon Fibre Reinforced PEEK Composite Plating System for Anterior Cervical Discectomy and Fusion

Helena Milavec [1,2,*], Christoph Kellner [1], Nivetha Ravikumar [2], Christoph E. Albers [1], Till Lerch [1], Sven Hoppe [1], Moritz C. Deml [1], Sebastian F. Bigdon [1], Naresh Kumar [2], and Lorin M. Benneker [1]

[1] Department of Orthopaedic Surgery, Spine Unit, Inselspital, Bern University Hospital, 3010 Bern, Switzerland
[2] Department of Orthopaedic Surgery, National University Health System (NUHS)–Tower Block, Level 11, 1E Kent Ridge Road, Singapore 119228, Singapore
* Correspondence: helena.milavec@insel.ch; Tel.: +41-31-664-04-40; Fax: +41-31-632-36-00

Table S1. Fusion rates based on indication and number of levels.

Fusion Rates	Indication Trauma	Degenerative	Level Treated Monosegmental	Bi-/Trisegmental
I (n = 22)	15	7	15	7
II (n = 6)	1	5	0	6
III (n = 7)	3	4	5	2

Table S2. Possible predisposing factors for nonunion.

	Fusion Grade III (Bridwell) (n = 7)
Sex	
Male	5
Female	2
Age	
<65	6
>65	1
Smoking	
No	6
Yes	1
Fusion level	
Mono	5
Bi-/trisegm.	2
BMI	
<25	6
>25	1
Diab. Mell.	
Yes	1
No	6
